# Medicine Shortages: Gaps Between Countries and Global Perspectives

**DOI:** 10.3389/fphar.2019.00763

**Published:** 2019-07-19

**Authors:** Angela Acosta, Egdda Patricia Vanegas, Joan Rovira, Brian Godman, Tomasz Bochenek

**Affiliations:** ^1^ISAGS, South American Institute of Government in Health, UNASUR, Rio de Janeiro, Brazil; ^2^RAM Group, National University of Colombia, Bogotá, Colombia; ^3^SEPRO Research Group, National University of Colombia, Bogotá, Colombia; ^4^Andalusian School of Public Health, Granada, Spain; ^5^Strathclyde Institute of Pharmacy and Biomedical Sciences, University of Strathclyde, Glasgow, United Kingdom; ^6^Division of Clinical Pharmacology, Karolinska Institute, Karolinska University Hospital Huddinge, Stockholm, Sweden; ^7^School of Pharmacy, Sefako Makgatho Health Sciences University, Garankuwa, South Africa; ^8^Department of Drug Management, Faculty of Health Sciences, Jagiellonian University Medical College, Krakow, Poland

**Keywords:** medicine shortages, medicine access, pharmaceutical policy, medicine supply, South America, Europe, North America, Western Asia

## Abstract

**Introduction:** Over the last decade, global health policies and different research areas have focused on the relevance and impact of medicine shortages. Published studies suggest there have been difficulties with access to medicines since the beginning of the 20th century, and there have been advances in our understanding and management of the problem since then. However, in view of global and regional health care concerns with shortages, we believe this phenomenon needs to be characterized and described more fully regarding the types of medicines affected, possible causes, and potential strategies to address these. The aim of this scoping review was to identify, compare if possible, and characterize the recent literature regarding the situation of medicines shortages between countries, and provide different perspectives, including a global context and national approaches.

**Methodology:** A scoping study presented as a narrative review of the situation and findings principally based on published articles.

**Results:** Based on the reported cases in the literature, a typology of medicines shortage and supply interruption episodes and their causes were proposed; national approaches to notify and manage the medicines shortages cases were described and classified by update frequency; principal differences between market and supply chain management perspectives of the situation were identified and global and countries’ perspectives were described.

**Conclusion:** Policy makers require solutions that prevent those cases in which the population’s health is affected by episodes of medicine shortages and/or interruption in the supply chain. There is also a need to generate a glossary related to logistics management and the availability of medicines which will be useful to understand and overcome shortages. In addition, recognize that potential solutions are not only related with actions linked to research, development and innovation, but much wider. Overall, we believe this article can act as a basis for future discussions in this important area.

## Introduction

The World Health Organization (WHO) defined “access to medicines” as a multidimensional problem in view of the rising prices of new medicines and persisting problems of medicine shortages among others (World Health Organization, 2018). Other concerns include out-of-pocket payments, which are especially important in lower- and middle-income countries (LMICs) where expenditure of medicines can be up to 70% of total health care expenditure and potentially catastrophic for patients and their families if they become ill (Cameron et al., 2009; Ofori-Asenso and Agyeman, 2016; World Health Organization, 2018). However, this is outside the scope of this article, which principally deals with issues of shortages of medicines.

The last WHO Director-General’s report included a variety of terms on global medicine shortages such as “shortage,” “scarcity” (only in the Spanish version), and “stock outs” (only in the English version) that demand a comprehensive approach across countries (World Health Organization, 2018). Consequently, there is an urgent need to develop a set of terminologies related to the problems identified by countries regarding the continual availability of essential medicines.

There are different situations leading to out of stock of medicines. Some of them can be solved without causing obstacles to health care provision or in the availability of the best therapeutic option, whereas others may require additional efforts and ways to overcome affected health conditions. There have been several important efforts to document countries’ experiences and potential ways forward to address concerns with medicine shortages. The study of ISAGS UNASUR published in 2017 characterized and analyzed the situation among eight South American countries: Bolivia, Chile, Colombia, Ecuador, Paraguay, Peru, Venezuela, and Uruguay (ISAGS, 2017). In 2018, Bochenek et al. (2018) systematically characterized, compared, and evaluated current measures, as well as legislative and organizational frameworks, to address medicines shortages among a wide range of European and Western Asian countries. These included 20 countries of the European Union (EU) and the European Free Trade Association (EFTA)—Austria, Belgium, Croatia, the Czech Republic, Estonia, France, Greece, Hungary, Ireland, Italy, Latvia, Lithuania, Malta, Norway, Poland, Portugal, Slovakia, Slovenia, Spain, and Switzerland. In addition, eight non-EU/EFTA countries: Albania, Azerbaijan, Israel, Kosovo, Montenegro, Republic of Srpska (Bosnia and Herzegovina), Serbia, and Turkey.

Both publications involving descriptions from different regions of the world with similar findings about formal definitions as well as strategies that had been developed to prevent or mitigate against medicine shortages. Both publications emphasized the urgent need to explore further the phenomena to identify mechanisms which introduce possible solutions for situations affected by medicine shortages, as well as facilitate the monitoring and assessing of signals and subsequent actions. However, there is a need to consolidate current findings to provide additional direction given current concerns, building on the experiences of other countries including Australia, Canada and the United States, as well as more recent research findings (Gupta and Huang, 2013; The Society of Hospital Pharmacists of Australian, SHPA, 2017; Videau et al., 2019).

Learning from other countries’ experiences should not be underestimated or underutilized in shaping local or national pharmaceutical policies (Godman et al., 2010; Gupta and Huang, 2013; Godman et al., 2014; Moon et al., 2014; Godman et al., 2015; Moorkens et al., 2017; The Society of Hospital Pharmacists of Australian, SHPA, 2017; Godman et al., 2018; Kwon et al., 2018; Videau et al., 2019). Lessons can be drawn from cross-country comparisons, even if a given country’s characteristics do not perfectly correspond in terms of geographical location, size, demography, economy, or type of health care system. Consequently, the objectives of this article are to consolidate current findings as well as characterize and describe more fully the situation across continents to provide further evidence about the types of affected medicines, identified causes, and potential strategies to address shortages. Subsequently, use the findings to provide suggestions to address this important topic.

## Materials and Methods

The design of this study is a scoping review. Scoping reviews have been useful to describe broad topic and provide an overview of diverse literature, including different study designs and methodologies, both widely available and gray scientific articles and reports (Pham et al., 2015).

Previous efforts have helped identify the main characteristics to describe the medicine shortage situation including potential definitions, general characteristics of the problem, description of information systems, potential and perceived causes of shortages, and implemented solutions (UNASUR, 2014; De Weerdt et al., 2015a; De Weerdt et al., 2015b; Pauwels et al., 2015; de Weerdt et al., 2017; Nurse-Findlay et al., 2017; Bochenek et al., 2018). We have built on this including the studies of Bochenek et al. (2018) and ISAGS (2017).

Full information on the content of the ISAGS survey form, including all the detailed questions which were posed in study on South American countries, can be found in the **Supplementary Material** to this paper. Likewise, detailed information on the content of the survey performed in European and Western Asian countries can be found in the **Supplementary Material** to the published study of Bochenek et al. Both surveys asked about existence of definitions of shortages of medicines, general characteristics of the problem, description of information systems, groups of medicines in shortage and particular molecules, potential and perceived causes of shortages, existence of processes, protocols and indicators to address shortages when they appear and to monitor their dynamics, and implemented solutions among others.

### Search Process

#### First Step

**Figure 1:**
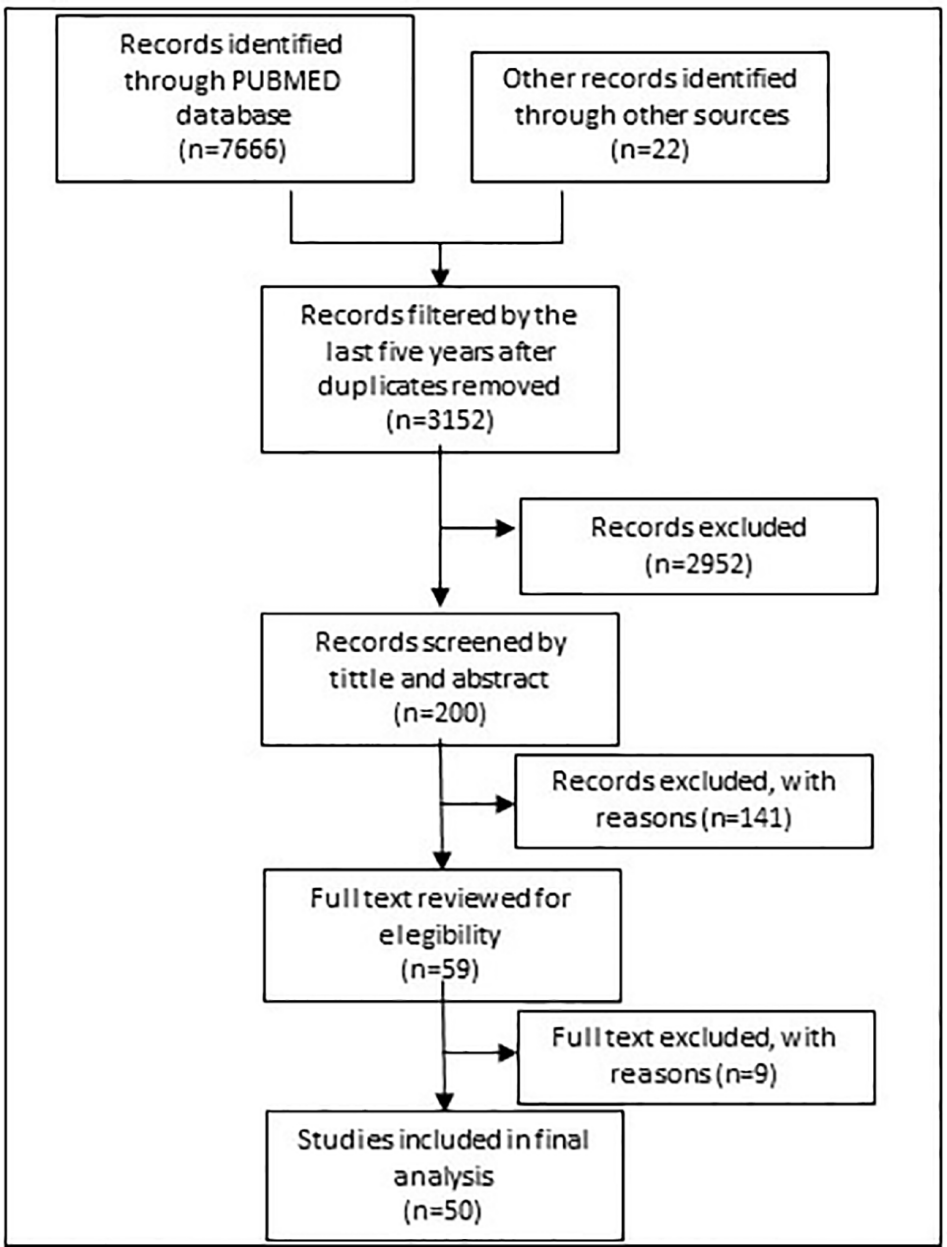
Flow chart of first literature review process.

**Figure 1** shows the search process. In 2017, eligible studies were first identified from a search of PubMed with the following general MeSH terms strategy “DRUG OR MEDICINE AND SHORTAGE.” The results were subsequently filtered by period (5 years from May 2012 to 2017), with references filtered by tittle and abstract taking into account representative descriptions of countries.

Other gray literature was retrieved from websites of the WHO, ministries of health and national health authorities. In addition, legal acts, as well as information gathered and disclosed within the public domain by organizations involved in pharmaceutical markets.

#### Second Step

**Figure 2:**
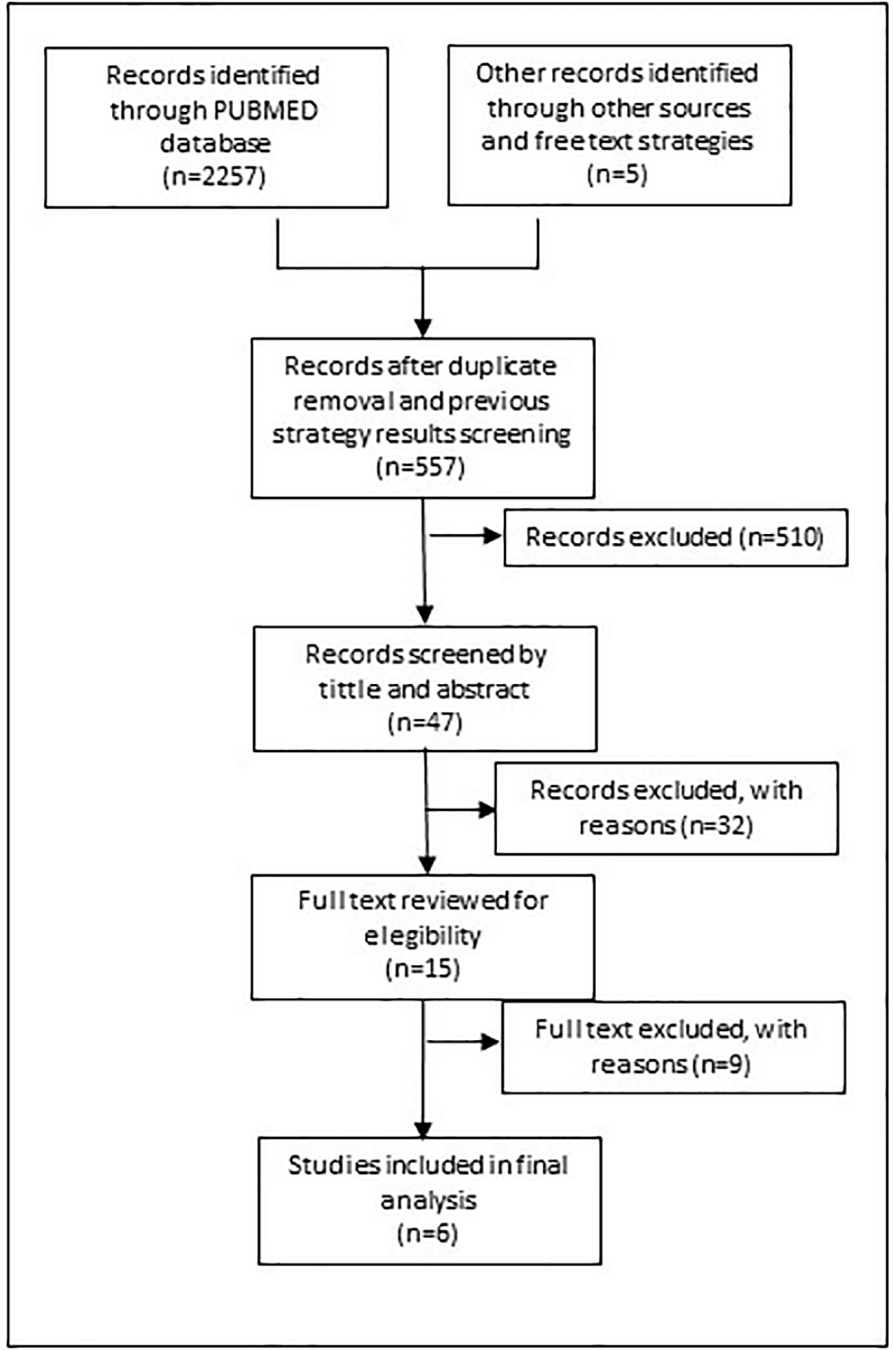
Flow chart of second literature review process.

**Figure 2** shows the search process for the update implemented in 2019. The same search strategy was used filtered by period (January 2016 until April 2019) to help recover additional eligible studies. The strategy was also translated into Portuguese and Spanish to run a search in Lilacs, a specific database for Latin America countries.

To retrieve other potential studies, free text search terms and a snowball literature review was undertaken. We ran the term “SHORTAGE” with specific countries and continents including Africa, Australia, Canada, China, Costa Rica, Japan, Mexico, Panama and the United Kingdom.

#### Inclusion Criteria

This review includes studies that describe the medicines shortage situation within a large jurisdiction or system of care, and settings could be regional, national, or international.

#### Selection Process

Two reviewers (EV, AA) performed the screening of abstracts based on the tittle/abstract review. The full text of potential eligible studies was retrieved if one or both authors thought they were relevant. Disagreements were resolved by discussion and, when necessary, including a third author (TB) in the discussion.

#### Data Extraction

Data extracted from each study included the authors and year of publication, jurisdictional level, existence of definitions, general characteristics of shortages, medicines involved, description of information systems including four categories of frequencies of shortages reports (high, medium, low, unspecified), potential and perceived causes of shortages, and possible implemented solutions at jurisdictional levels.

## Results

### Description of Studies

By 2017, the literature review had identified 50 references to include in this scoping review, and by 2019 another 6 new references where identified. Of these 56 references, 9 studies had a regional broad focus for Europe and Latin America. We found studies with country description of medicines shortage for one East Asia country (China), 7 European countries (Belgium, Finland, France, Ireland, Slovenia, Spain and the United Kingdom), two Latin America countries (Brazil and Venezuela); two North American countries (Canada and the United States); two Oceania countries (Australia and Fiji); and 4 Western Asia countries (Iran, Iraq, Jordan and Israel) (**Table 1**).

**Table 1:** Distribution of included references by geography.

Region/country	Number	Ref	Total by region
**East Asia**			**1**
China	1	• Yang, C., Wu, L., Cai, W., Zhu, W., Shen, Q., Li, Z., et al. (2016). Current situation,determinants, and solutions to drug shortages in Shaanxi Province, China: a qualitative study. PLoS ONE 11: e0165183. doi: 10.1371/journal.pone.0165183 (Yang et al., 2016)	
**Europe**			**16**
Europe (broad focus)	8	• Bochenek T, Abilova V, Alkan A, Asanin B, Beriain I de M, Besovic Z, et al. Systemic measures and legislative and organizational frameworks aimed at preventing or mitigating drug shortages in 28 European and Western Asian Countries. Front Pharmacol. 2018;8(JAN). (Bochenek et al., 2018) • Pauwels, K., Huys, I., Casteels, M., and Simoens, S. (2014). Drug shortages in European countries: a trade-off between market attractiveness and cost containment? BMC Health Serv Res. 14:438. doi: 10.1186/1472-6963-14-438 (Pauwels et al., 2014) • Pauwels, K., Simoens, S., Casteels, M., and Huys, I. (2015). Insights into European drug shortages: a survey of hospital pharmacists. PLoS ONE 10:e0119322.doi: 10.1371/journal.pone.0119322 (Pauwels et al., 2015) • Birgli, A. G. (2013). An Evaluation of Medicines Shortages in Europe with More In-Depth Review of These in France, Greece, Poland, Spain and the United Kingdom. Available online at: https://www.eaepc.org/images/pdf/evaluation.pdf (Birgli® ag, 2013) • Bogaert, P., Bochenek, T., Prokop, A., and Pilc, A. (2015). A qualitative approach to a better understanding of the problems underlying drug shortages, as viewed from Belgian, French and the European Union’s perspectives. PLoS ONE 10:e0125691. doi: 10.1371/journal.pone.0125691 (Bogaert et al., 2015) • De Weerdt, E., Simoens, S., Casteels, M., and Huys, I. (2017b). Clinical, economic and policy implications of drug shortages in the European union. Appl. Health Econ. Health Policy 15, 441–445. doi: 10.1007/s40258-016-0264-z. (de Weerdt et al., 2017) • De Weerdt, E., Simoens, S., Hombroeckx, L., Casteels, M., and Huys, I. (2015b).Causes of drug shortages in the legal pharmaceutical framework. Regul. Toxicol.Pharmacol. 71, 251–258. doi: 10.1016/j.yrtph.2015.01.005 (De Weerdt et al., 2015b) • De Weerdt E, Simoens S, Casteels M, Huys I. Toward a European definition for a drug shortage: A qualitative study. Front Pharmacol. 2015;6(OCT):1–9. (De Weerdt et al., 2015a)	
Belgium	1	• Bauters T, Claus BO, Norga K, Huys I, Simoens S, Laureys G. Chemotherapy drug shortages in paediatric oncology: A 14-year single-center experience in Belgium. J Oncol Pharm Pract [Internet]. 2016;22(6):766–70. Available from: http://opp.sagepub.com/cgi/doi/10.1177/1078155215610915 (Bauters et al., 2016)	
Finland	1	• Heiskanen, K., Ahonen, R., Kanerva, R., Karttunen, P., and Timonen, J. (2017). The reasons behind medicine shortages from the perspective of pharmaceutical companies and pharmaceutical wholesalers in Finland. PLoS ONE 12:e0179479.doi: 10.1371/journal.pone.0179479. (Heiskanen et al., 2017)	
France	1	• Bocquet, F., Degrassat-Théas, A., Peigné, J., and Paubel, P. (2017). The new regulatory tools of the 2016 Health Law to fight drug shortages in France. Health Policy 121, 471–476. doi: 10.1016/j.healthpol.2017.03.007 (Bocquet et al., 2017)	
Ireland	1	• Kavanagh J. (2017). How Pharmaceutical Supply Chains Can Be Managed to Minimise the Number of Medicines Shortages, Unpublished Master’s thesis, University College Dublin, Ireland. (Kavanagh, 2017) • Vella Bonanno, P., and Gavril, F. (2011). Seven years of EU Pharmaceutical regulation in Malta. WHO Drug Inf. 25, 341–412. (Bonnanno and Gavril, 2011)	
Slovenia	1	• Pfeffer, K., and Mozolová, B. (2017). Re-export of drugs in the Slovak Republic vol. 2 – the Act [Internet]. Available: https://www.twobirds.com/en/news/articles/2017/uk/ils/re-export-of-drugs-in-the-slovak-republic-vol-2-the-act (Pfeffer and Mozoľová, 2017)	
Spain	1	• Servicio Vasco de Salud -Osakidetza. Desabastecimiento De Medicamentos: Un Problema Sin Resolver [Internet]. Vol. 23, INFORMACIÓN FARMACOTERAPÉUTICA DE LA COMARCA. Vitoria - Gasteiz; 2016. Available from: http://www.osakidetza.euskadi.eus/contenidos/informacion/cevime_infac_2015/es_def/adjuntos/INFAC_Vol_23_N_7_Desabastecimientos.pdf (Servicio Vasco de Salud –Osakidetza, 2016)	
United Kingdom	2	• Costelloe, E. M., Guinane, M., Nugent, F., Halley, O., and Parsons, C. (2014). An audit of drug shortages in a community pharmacy practice. Ir. J. Med. Sci.435–440. doi: 10.1007/s11845-014-1139-7 (Costelloe et al., 2015) • Group AP. Why drug shortages occur. Drug Ther Bull [Internet]. 2015;53(3):33–6. Available from: https://dtb.bmj.com/content/53/3/33.full (All-party Pharmacy, 2015)	
**Latin America**			**3**
Latin America (broad focus)	1	• ISAGS. Situation of Essential Medicines at Risk of Supply Shortage with Emphasis on South American Countries [Internet]. Rio de Janeiro; 2017. Available from: http://isags-unasur.org/en/publicacao/situation-of-essential-medicines-at-risk-of-supply-shortage-with-emphasis-on-south-american-countries-2/ (ISAGS, 2017)	
Brazil	1	• Perini E, Rosa MB, Reis AMM, Perini E. Drug shortage: a public health problem. Cad Saude Publica [Internet]. 2016 Oct [cited 2017 Jul 10];32(10). Available from: http://www.scielo.br/scielo.php?script=sci_arttext&pid=S0102-311X2016001000301&lng=en&tlng=en (Rosa et al., 2016)	
Venezuela	1	• Aular de González Y. Escasez de medicamentos y su repercusión en la salud. Salus. 2014;18(2):5–6. (Aular de González, 2014)	
**North America**			**29**
Canada	3	• Morrison, A. (2011). Drug Supply Disruptions [Environmental Scan Issue 17]Ottawa: Canadian Agency for Drugs and Technologies in Health. Available online at: https://www.cadth.ca/drug-supply-disruptions (Morrison, 2011) • Kaposy C. Drugs, money, and power: the Canadian drug shortage. J Bioeth Inq [Internet]. 2014 Mar [cited 2014 May 29];11(1):85–9. Available from: http://www.ncbi.nlm.nih.gov/pubmed/24357073 • Videau M, Lebel D, Bussières JF. Drug shortages in Canada: Data for 2016–2017 and perspectives on the problem. Ann Pharm Fr [Internet]. 2019; Available from: https://doi.org/10.1016/j.pharma.2018.11.007 (Videau et al., 2019)	
United States	26	• Schweitzer, S. O. (2013). How the US Food and Drug Administration can solve the prescription drug shortage problem. Am. J. Public Health. 10310–14. doi: 10.2105/AJPH.2013.301239 (Schweitzer, 2013) • Ventola, C. L. (2011). The drug shortage crisis in the United States: causes, impact, and management strategies. P T. 36, 740–757. (Ventola, 2011) • Goldsack, J. C., Reilly, C., Bush, C., McElligott, S., Bristol, M. N., Motanya, U. N., et al. (2014). Impact of shortages of injectable oncology drugs on patient care. Am. J. Heal. Pharm. 71, 571–578. doi: 10.2146/ajhp130569 (Goldsack et al., 2014) • Butterfield, L., Cash, J., and Pham, K. (2015). Position statement drug shortages and implications for pediatric patients. J. Pediatr. Pharmacol. Ther. 20, 149–152. doi: 10.5863/1551-6776-20.2.149 (Butterfield et al., 2015) • Mazer-Amirshahi, M., Goyal, M., Umar, S. A., Fox, E. R., Zocchi, M., Hawley, K.L., et al. (2017). U.S. drug shortages for medications used in adult critical care(2001-2016). J. Crit. Care. 41, 283–288. doi: 10.1016/j.jcrc.2017.06.005 (Mazer-Amirshahi et al., 2017) • McLaughlin,M.M., and Skoglund, E.W. (2015). Drug shortages and patient safety. J. Infus. Nurs. 38, 205–208. doi: 10.1097/NAN.0000000000000101 • McLaughlin, M., Kotis, D., Thomson, K., Harrison, M., Fennessy, G., Postelnick, M., et al. (2013). Effects on patient care caused by drug shortages: a survey. J. Manag. Care Pharm. 19, 783–788. doi: 10.18553/jmcp.2013.19.9.783 • McKeever AE, Bloch JR, Bratic A. Drug shortages and the burden of access to care: A critical issue affecting patients with cancer. Clin J Oncol Nurs. 2013; (McKeever et al., 2013) • Alevizakos M, Detsis M, Grigoras CA, Machan JT, Mylonakis E. The Impact of Shortages on Medication Prices: Implications for Shortage Prevention. Drugs. 2016;76(16):1551–8 (Alevizakos et al., 2016) • Griffith MM, Gross AE, Sutton SH, Bolon MK, Esterly JS, Patel JA, et al. The impact of anti-infective drug shortages on hospitals in the United States: Trends and causes. Clin Infect Dis. 2012;54(5):684–91. (Griffith et al., 2012) • Steers WD. Falling short: Causes and Implications of Drug Shortages in the United States. J Urol [Internet]. 2014;192(5):1315–7. Available from: http://www.ncbi.nlm.nih.gov/pubmed/25218647 (Steers, 2014) • Gabrielli A, Layon NT, Bones HL, Layon AJ. The Tragedy of the Commons - Drug Shortages and Our Patients’ Health. Am J Med [Internet]. 2016;129(12):1237–8. Available from: http://linkinghub.elsevier.com/retrieve/pii/S0002934316310130%0A http://www.ncbi.nlm.nih.gov/pubmed/28029357 (Gabrielli et al., 2016) • Rinaldi F, de Denus S, Nguyen A, Nattel S, Bussières J-F. Drug Shortages: Patients and Health Care Providers Are All Drawing the Short Straw. Can J Cardiol [Internet]. 2016; Available from: http://linkinghub.elsevier.com/retrieve/pii/S0828282X1630842X%0A http://www.ncbi.nlm.nih.gov/pubmed/27923583 (Rinaldi et al., 2017) • Parsons HM, Schmidt S, Karnad AB, Liang Y, Pugh MJ, Fox ER, et al. Association Between the Number of Suppliers for Critical Antineoplastics and Drug Shortages: Implications for Future Drug Shortages and Treatment. J Oncol Pract [Internet]. 2016;12(3):249–50. Available from: http://jop.ascopubs.org/cgi/doi/10.1200/JOP.2015.007237 • Chen SI, Fox ER, Kennedy Hall M, Ross JS, Bucholz EM, Krumholz HM, et al. Despite federal legislation, shortages of drugs used in acute care settings remain persistent and prolonged. Health Aff. 2016;35(5):798–804. (Chen et al., 2016) • Fox ER, Tyler LS. Potential Association between Drug Shortages and High-Cost Medications. Pharmacotherapy [Internet]. 2016; Available from: http://www.ncbi.nlm.nih.gov/pubmed/27891635 (Fox and Tyler, 2017) • Warkentin J, Flood J, Kanouse J, Shah N, Cronin A. Impact of a Shortage of First-Line Antituberculosis Medication on Tuberculosis Control — United States, 2012–2013. Morb Mortal Wkly Rep - CD [Internet]. 2013;62(20):396–400. Available from: http://www.ncbi.nlm.nih.gov/pubmed/23698603 • Jagsi R., Spence R., Rathmell W.K., Bradbury A., Peppercorn J., et al. Ethical considerations for the clinical oncologist in an era of oncology drug shortages. Oncologist. 2014;19(2):186–92. (Jagsi et al., 2014) • Becker DJ, Talwar S, Levy BP, Thorn M, Roitman J, Blum RH, et al. Impact of oncology drug shortages on patient therapy: unplanned treatment changes. J Oncol Pract. 2013;9(4):122–8. (Becker et al., 2013) • Gupta DK, Huang S-M. Drug Shortages in the United States: A Critical Evaluation of Root Causes and the Need for Action. Clin Pharmacol Ther [Internet]. 2013;93(2):133–5. Available from: http://www.ncbi.nlm.nih.gov/pubmed/23337520 (Gupta and Huang, 2013)	
		• Caulder C, Mehta B, Bookstaver P, Sims L, Stevenson B, South Carolina Society of Health-Sy. Impact of Drug Shortages on Health System Pharmacies in the Southeastern United States. Hosp Pharm [Internet]. 2015;50(4):279–86. Available from: https://www.ncbi.nlm.nih.gov/pmc/articles/PMC4589883/pdf/hpj-50-279.pdf (Caulder et al., 2015) • Golembiewski J. Drug shortages in the perioperative setting: causes, impact, and strategies. J Perianesth Nurs [Internet]. 2012 Aug [cited 2013 Mar 7];27(4):286–92. Available from: http://www.ncbi.nlm.nih.gov/pubmed/22828028 (Golembiewski, 2012) • Daley M, Lat I, Kane-Gill S. Applicability of Guideline Recommendations Challenged in the Setting of Drug Shortages. Crit Care Med [Internet]. 2013;41(7):e142–3. Available from: http://content.wkhealth.com/linkback/openurl?sid=WKPTLP:landingpage&an=00003246-201307000-00058 • McLaughlin M, Kotis D, Thomson K, Harrison M, Fennessy G, Postelnick M, et al. Effects on Patient Care Caused by Drug Shortages: A Survey. J Manag Care Pharm [Internet]. 2013;19(9):783–8. Available from: http://www.jmcp.org/doi/10.18553/jmcp.2013.19.9.783 (McLaughlin et al., 2013) • Administra- D, Act I, Hoffman RS. Antidote shortages in the United States: impact and response. Clin Toxicol (Phila) [Internet]. 2014;52(3):157–9. Available from: http://www.ncbi.nlm.nih.gov/pubmed/24397753 (American College of Medical Toxicology, American Academy of Clinical Toxicology, 2015) • Bible JR, Evans DC, Payne B, Mostafavifar L. Impact of Drug Shortages on Patients Receiving Parenteral Nutrition After Laparotomy. J Parenter Enter Nutr [Internet]. 2014;38(2_suppl):65S–71S. Available from: http://journals.sagepub.com/doi/10.1177/0148607114550317	
**Oceania**			**2**
Australia	1	• The Society of Hospital Pharmacists of Australian, SHPA. Medicine shortages in Australia. A snapshot of shortages in australian hospitals. Victoria; 2017. (The Society of Hospital Pharmacists of Australian, SHPA, 2017)	
Fiji	1	• Walker, J., Chaar, B. B., Vera, N., Pillai, A. S., Lim, J. S., Bero, L., et al. (2017). Medicine shortages in Fiji: a qualitative exploration of stakeholders’ views. PLoS ONE 12:e0178429. doi: 10.1371/journal.pone.0178429 (Walker et al., 2017)	
**Western Asia**			**4**
Iran	1	• Setayesh S, Mackey TK. Addressing the impact of economic sanctions on Iranian drug shortages in the joint comprehensive plan of action: promoting access to medicines and health diplomacy. Global Health [Internet]. 2016;12(1):31. Available from: http://globalizationandhealth.biomedcentral.com/articles/10.1186/s12992-016-0168-6 (Setayesh and Mackey, 2016)	
Iraq	1	• Cousins S. Iraq: staff and medicine shortages are major challenges. Lancet [Internet]. 2014;384(9947):943–4. Available from: http://linkinghub.elsevier.com/retrieve/pii/S0140673614616159 (Cousins, 2014)	
Jordan	1	• Awad, H., Al-Zu’bi, Z.M. F., and Abdallah, A. B. (2016). A quantitative analysis of the causes of drug shortages in Jordan: a supply chain perspective. Int. Bus. Res.9:53. doi: 10.5539/ibr.v9n6p53 (Awad et al., 2016)	
Israel	1	• Schwartzberg E, Ainbinder D, Vishkauzan A, Gamzu R. Drug shortages in Israel: regulatory perspectives, challenges and solutions. Isr J Health Policy Res [Internet]. 2017;6(1):17. Available from: http://ijhpr.biomedcentral.com/articles/10.1186/s13584-017-0140-9%0A http://www.ncbi.nlm.nih.gov/pubmed/28392910%0A http://www.pubmedcentral.nih.gov/articlerender.fcgi?artid=PMC5376685 (Schwartzberg et al., 2017)	
**Cross settings comparisons between countries**			**1**
The United States and Arabia Saudi – Hospital Setting	1	• Alsheikh, M., Seoane-Vazquez, E., Rittenhouse, B., Fox, E. R., and Fanikos, J. (2016). A comparison of drug shortages in the Hospital Setting in the United States and Saudi Arabia: an exploratory analysis. Hosp. Pharm. 51,370–375. doi: 10.1310/hpj5105-370 (Alsheikh et al., 2016)	

Three of the identified articles had developed surveys addressed to the ministries of health, state medicines agencies and local health authorities of various countries from three regions to discuss potential ways to address medicine shortages (Bogaert et al., 2015; ISAGS, 2017; Bochenek et al., 2018). One study described the literature review in terms of the characteristics of shortages and country definitions of shortages (De Weerdt et al., 2015a). Another regional study described the shortage situation from the perspective of hospital pharmacists and prescribers (Pauwels et al., 2015).

The United States (US) is the country with more publications in the field (26 references) than many other countries. Many of these publications describe the professionals’ perspective of the situation at the health care jurisdictional level (Morrison, 2011; Golembiewski, 2012; Griffith et al., 2012; Becker et al., 2013; McKeever et al., 2013; McLaughlin et al., 2013; Bible et al., 2014; Goldsack et al., 2014; Butterfield et al., 2015; Caulder et al., 2015; McLaughlin and Skoglund, 2015; Gabrielli et al., 2016; Parsons et al., 2016; Setayesh and Mackey, 2016; Bocquet et al., 2017; Fox and Tyler, 2017; Mazer-Amirshahi et al., 2017; Rinaldi et al., 2017; Schwartzberg et al., 2017). Another study performed a comparison of medicines shortages between two hospital settings, one from Arabia Saudi and the other from the US (Alsheikh et al., 2016).

### Contexts and Reasons of Medicines Shortage

The description of the shortage situations may be associated with 4 principal causes or determinants, namely: market, supply chain management, manufacturing, and political issues (**Table 2**).

**Table 2:** Most frequent reasons for medicine shortages.

Category	Cause
Market	Increase in sales (McKeever et al., 2013; Ordre National des Pharmaciens, 2015) Price-related aspects (McKeever et al., 2013; Ordre National des Pharmaciens, 2015; Alevizakos et al., 2016; Yang et al., 2016) Voluntary withdrawal (Griffith et al., 2012; Steers, 2014; Chen et al., 2016; Yang et al., 2016; Rinaldi et al., 2017) Unexpected increases and unexpected changes in clinical practice (Griffith et al., 2012; Yang et al., 2016) Parallel or gray markets (Yang et al., 2016) Loss of market interest (Videau et al., 2019) Relocation of production facilities (Videau et al., 2019) Speculation in international markets (Videau et al., 2019) Mergers of manufacturers and joint purchasing group (Videau et al., 2019)
Supply chain management	Structure of the network or supply chain in the country (Schwartzberg et al., 2017) Supply of raw materials and excipients (Griffith et al., 2012; Ordre National des Pharmaciens, 2015; Chen et al., 2016; Gabrielli et al., 2016; Parsons et al., 2016; Bocquet et al., 2017; Rinaldi et al., 2017; Schwartzberg et al., 2017)
Manufacturing process	Quality concerns (Griffith et al., 2012; Gabrielli et al., 2016; Bocquet et al., 2017; De Weerdt et al., 2017; Rinaldi et al., 2017; Schwartzberg et al., 2017) Changes in the product formulation (Yang et al., 2016; Rinaldi et al., 2017) Industrial development capacities (Steers, 2014; Gabrielli et al., 2016; Yang et al., 2016; Fox and Tyler, 2017) Production problems (Videau et al., 2019)
Political and ethical issues	Regulatory problems (McKeever et al., 2013; Chen et al., 2016; Gabrielli et al., 2016; Parsons et al., 2016; Yang et al., 2016; Bocquet et al., 2017; Schwartzberg et al., 2017). Public policy (Cousins, 2014; Setayesh and Mackey, 2016; French Parliament). Social conflicts (Setayesh and Mackey, 2016; Bochenek et al., 2018). Enhancement of the legal and normative frameworks applicable to the manufacture of medicines (Videau et al., 2019)

Some authors have concluded that these categories are interrelated and have one aspect in common, that is, many cases are related to the availability of safe and effective medicines with low profitability or with low sales making them non-viable commercially (McKeever et al., 2013; Alevizakos et al., 2016; Schwartzberg et al., 2017).

In South American countries, medicine shortages generally occur with mature products without suppliers in the market due to lack of market viability, and correspond mostly to parenteral medicines with low profitability (ISAGS, 2017).

Some countries issue alerts about medicines that are simply not available on the market in their country even if there is enough money to pay for them within their health care systems (Bochenek et al., 2018).

Finally, Bochenek et al. (2018) addressed an increasing amount of evidence where medicines were unavailable in countries even if the products complied with current regulations and were financed within the health care system. As a result, these situations show that ethical and political issues could be affecting the timely availability of first-line therapeutic alternatives. These situations threaten the ability of clinicians and governments to fulfill their moral obligations to patients and society to provide benefit to patients, minimize harm, and promote equity.

Published studies in hospitals allow a better follow up of the health consequences of medicine shortages (Pauwels et al., 2015; de Weerdt et al., 2017); which is more difficult in ambulatory care (Golembiewski, 2012; Becker et al., 2013; Goldsack et al., 2014; Jagsi et al., 2014; Chen et al., 2016). This scoping review identified a number of published articles describing inpatient challenges regarding medicine shortages in the US (Morrison, 2011; Golembiewski, 2012; Griffith et al., 2012; Becker et al., 2013; McKeever et al., 2013; McLaughlin et al., 2013; Bible et al., 2014; Goldsack et al., 2014; Butterfield et al., 2015; Caulder et al., 2015; McLaughlin and Skoglund, 2015; Gabrielli et al., 2016; Parsons et al., 2016; Setayesh and Mackey, 2016; Bocquet et al., 2017; Fox and Tyler, 2017; Mazer-Amirshahi et al., 2017; Rinaldi et al., 2017; Schwartzberg et al., 2017).

### Medicines Involved

The characterization of supply shortages, their frequency, and the main groups of medicines affected among countries and regions are described in **Table 3**. The same characterization though is not made for the impact of shortages on patients’ health and health care systems. Descriptions in this respect generally correspond to descriptions of cases regarding either the impact of shortages on health conditions or medical specialties, or limitations in obtaining data and estimates made from surveys.

**Table 3:** Description of medicines classes with shortages in the selected countries.

Region/country*	Nervous system	Cardiovascular system	Anti-infectives systemic use	Cancer	Genitourinary system and sex hormones	Alimentary track and metabolism
**South America 2017**	17%	9%	21%	10%	7%	Non available
**Belgium 2009–2013**	23%	21%	11%	9%	Non available	8%
**Israel 2013–2015**	21%	15%	16%	Non available	8%	7%
**US 2013–2017**	18%	11%	Non available	9%	Non available	Non available
**Canada** (Videau et al., 2019)	31.8%	21.9%	8.5%	5.1%	Non available	0.1%
**Australia** (The Society of Hospital Pharmacists of Australian, SHPA, 2017)	12% (Anaesthetics) 9% (Neurology)	10%	20%	9.5%	10%	Non available
**China** (Yang et al., 2016)	13%	6%	6%	5.7%	11%	9%

**Figure 3:**
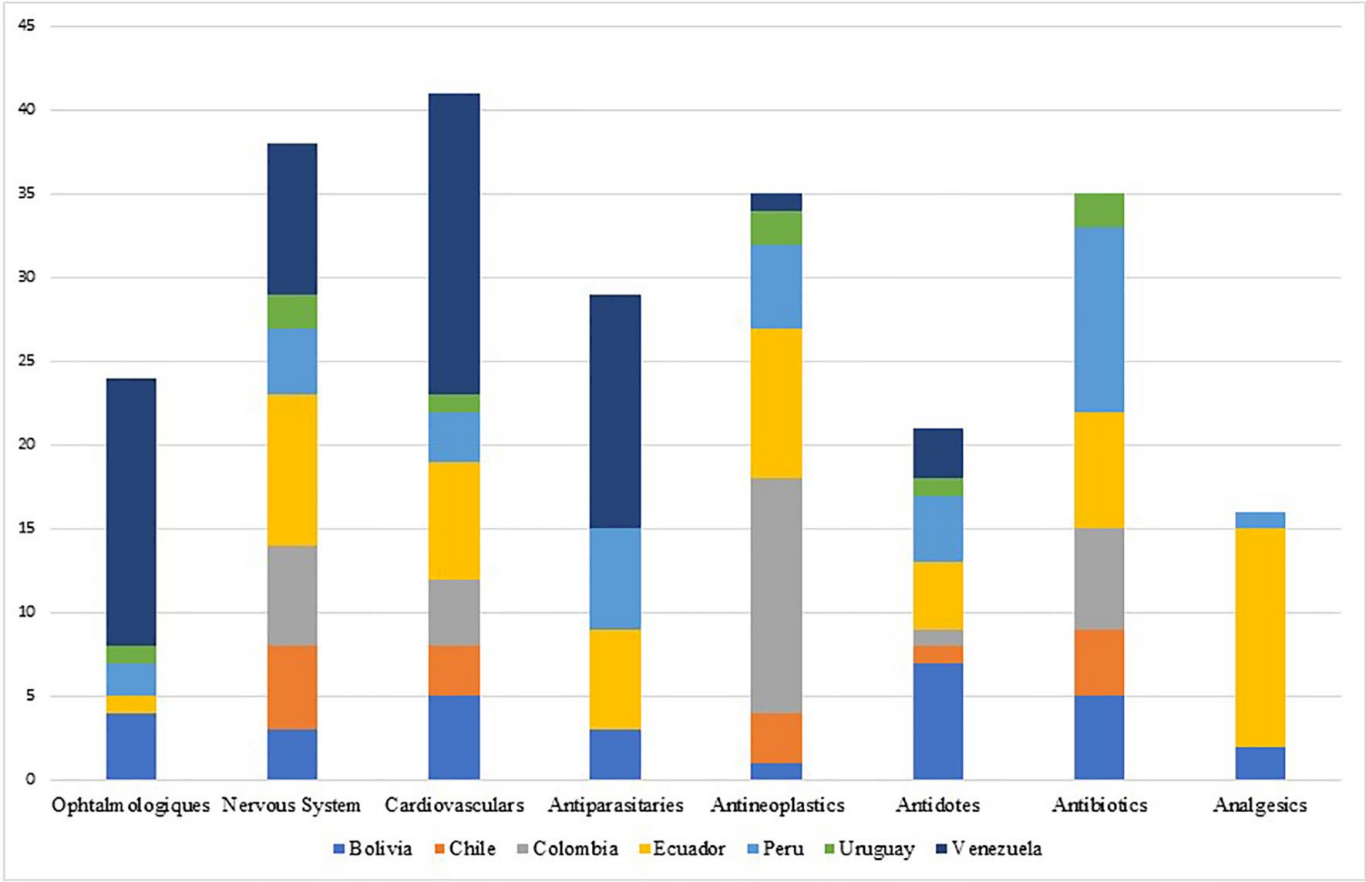
Principal medicine groups experiencing shortages among South American countries.

The shortage of essential medicines, including the active ingredients mostly used in injectable chemotherapy medicines, antibiotics, and anesthesia, is causing growing concern across regions including Europe, North America, Asia, and South America. However, the problem is much broader, affecting other classes of medicines - mainly parenteral medicines (Becker et al., 2013; Gulbis et al., 2013; Gupta and Huang, 2013; Jagsi et al., 2014; Caulder et al., 2015; De Weerdt et al., 2015b; De Weerdt et al., 2017; Schwartzberg et al., 2017) including anesthetics, nutrition and electrolyte solutions, enzyme replacement medicines, radiopharmaceuticals and antibiotics. The shortage of medicines has also been observed and documented for instance in Australia, Canada, China and Israel (Gray and Manasse, 2012; Kaposy, 2014; Schwartzberg et al., 2017). Most of the published evidence from countries of South America shows that shortages can occur even with essential medicines and especially injectable forms (ISAGS, 2017) (see **Figure 3**).

The groups of medicines, which were most frequently reported to be in shortage, were the cardiovascular medicines in Canada and Belgium; nervous system medicines in Australia, China, and Israel; and anti-infective medicines for most of the countries in South America (Bogaert et al., 2015; Yang et al., 2016; ISAGS, 2017; Schwartzberg et al., 2017; Videau et al., 2019).

**Figure 4:**
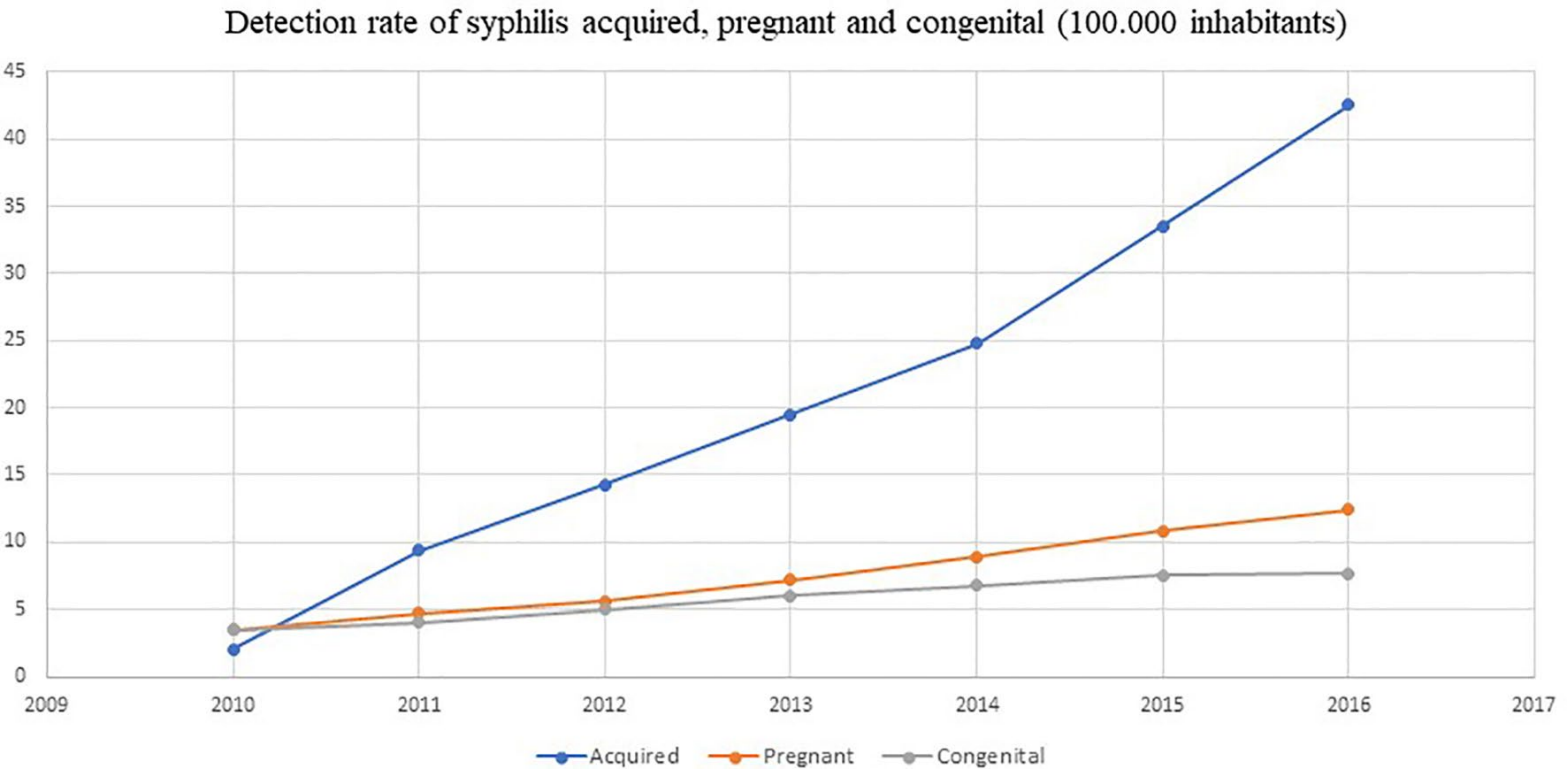
Detection rate of syphilis and congenital syphilis in Brazil. Source: Brazil Ministry of Health.

Brazil has reported the national shortage of penicillins (first-line treatment) as a result of the lack of specific raw materials for their production in the international market. This episode was described because of the increased incidence of syphilis, including also congenital syphilis, during the last 5 years and the concern this causes (see **Figure 4**). The alternative second-line antibiotics to treat acquired syphilis and the partners of pregnant women in Brazil, i.e., doxycycline and ceftriaxone, have doses of between 8 and 15 days, and there are concerns with adherence in practice (Lazarini and Barbosa, 2017).

In France (Bocquet et al., 2017), supply shortages with 71 types of medicine were identified. According to the ATC classification (Norwegian Institute of Public Nealth, 2018), the most prevalent medicine were antibiotics, oncological medicines, antidepressants, antipsychotics, tuberculosis medicines, vaccines, and immunoglobulins.

In Iran, at least 73 cases of medicine shortages were identified, among which 44% were essential. Potentially, they impacted on successful management of disease areas including HIV, epilepsy, hemophilia, thalassemia, and patients undergoing organ transplants (Setayesh and Mackey, 2016).

### Definitions

#### National Definitions

**Table 4** contains definitions adopted by countries, considering attributes with the formulation and agreement of potential definitions.

**Table 4:** Countries definitions.

Country	Terms/concepts	Definition
Argentina	Medicines lacking	“known circumstances or facts that could jeopardize the supply of products and cause their temporary or definitive discontinuity in the market”1
Australia	Shortage of a medicine	“There is a shortage of a medicine in Australia at a particular time if, at any time in the 6 months after that particular time, the supply of that medicine in Australia will not, or will not be likely to, meet the demand for the medicine for all of the patients in Australia who take, or who may need to take, the medicine”2 (The Society of Hospital Pharmacists of Australian, SHPA, 2017)
Belgium	Unavailability of medicines on the Belgian market	“A drug is unavailable when enterprises that are responsible for the marketing of the drug are unable to deliver that drug for an uninterrupted period of four consecutive days to the community pharmacies, hospital pharmacies or wholesalers in Belgium.” (De Weerdt et al., 2015a)
Brazil	“Temporary discontinuation” “Definitive discontinuation” “Unplanned discontinuation of the manufacture or importation of medicines”	of the manufacture or importation of medicines means that the license holder does not intend to cancel or does not intend to require the renewal of the registration of the product. of a product, in its turn, happens when the license holder intends to cancel or not to request the renewal of the registration of the product. Are those cases where quality, efficacy or safety properties of medicines are affected and may lead to a supply shortage in the market. (ISAGS, 2017)
Colombia	Medicines shortage	A situation which there is not enough supply to satisfy the demand of any medicine approved and marketed in at the country. (INVIMA, 2018)
Canada	Drug shortage	A situation in which an authorization holder for a drug is unable to meet the demand for the drug. Drug shortages can include temporary or permanent discontinuances in the production and supply of a drug (Videau et al., 2019)
Croatia	Disturbance on the medicines’ market	Not specific.
France	Drug shortage	A drug shortage is defined by law in France as an inability for a community pharmacy or a hospital pharmacy to deliver a drug within 72 h (Ministre des affaires sociales, Ministre de la santè, 2012). Additionally, drug shortages in France have been classified formally into two separate contexts of either stock or supply problems. A stock-related shortage is defined as the lack of possibility to manufacture a medicine, whereas a supply-related shortage is defined as a problem in the distribution chain that makes the supply of a medicine impossible, even if enough of the medicine has been manufactured (De Weerdt et al., 2015a).
Greece	Actual shortages Temporary interruptions in supply	“Pertains to the lack of capability to fulfill the demand and the non-availability of a drug in the whole health care system, without the possibility to obtain that medicine from any source.” “It refers to situations when drugs are not commercially available,” mainly for commercial reasons, for a limited time duration (Bochenek et al., 2018).
Hungary	“Drug shortage”	As a term is reported to be widely used in the legislation, to report in case they are not able to supply, but without any association with a concrete formal definition (Bochenek et al., 2018).
Italy	Short supply	The Italian Medicines Agency (AIFA) defines medicines in short supply as: “Medicines which are not available or not to be found in the whole Italian market, because the marketing authorization holder (MAH) is unable to guarantee the correct and regular supply to meet patients’ needs.” (Bochenek et al., 2018).
Norway	Temporary disruption of a medicine’s marketing	It is de facto considered to be a shortage as soon as it lasted for at least 2 weeks (Bochenek et al., 2018).
Peru	“Supply shortage” Unavailable Pharmaceutical Products Pharmaceutical product with limited supply	Considered as an operational definition in the management of drug availability indicators employed by the public bodies within the Ministry of Health and Regional Governments. Essential pharmaceutical products not supplied in the national market. Product with limited provision in the pharmaceutical market, which could generate access problems (availability and affordability) for the population
Spain	Supply problem	The Spanish Agency for Medicines and Health Products (Spanish acronym: AEMPS), being part of the Spanish Ministry of Health Care, defined the “supply problem” as a situation in which the number of available units of a drug in the pharmaceutical trade channel is below the level of national or local consumption needs, being often due to problems in the manufacturing or distribution of a drug
United States	Medicines shortages	“A period when the demand or projected demand for a medically necessary drug in the United States exceeds its supply” (Center for Drug Evaluation and research 2018).
Uruguay	Declaration of interruption of sale Interruption of marketing	Interruptions lasting 30 days or more. Due to exceptional interruptions by the manufacturer lasting less than 30 days, which must be communicated to and validated by the Department of Medicines. (ISAGS, 2017)

^1^ Administración Nacional de Medicamentos, Alimentos y Tecnología Médica. https://www.argentina.gob.ar/anmat

^2^ The Therapeutic Goods Administration. Medicine Shortages Information Initiative Available from: https://apps.tga.gov.au/prod/MSI/search#furtherinformation

One of the most important findings of this scoping review is that consulted sources, literature and institutional websites, have identified a considerable number of countries that are introducing legislative actions to cope the medicines shortages and had included formal definitions or related terms of medicine shortages.

Recently, Bochenek et al (2018) identified four countries in Europe with specific definitions, and ISAGS in 2017 (ISAGS, 2017) described three definitions for Latin American countries. This scoping review found progress in both regions, i.e., eight European countries and seven Latin America now have national medicines shortage definitions. In addition, by using free terms in the search strategies, we found definitions for one more region, North America (Canada and the United States), and for one country, Australia (**Table 4**). There are two primary sources of definitions: one from health authority agency websites (Argentina, Australia, Brazil, Colombia, Spain, US, Uruguay, Norway), and the other is the published literature for countries including Belgium, Canada, France, Greece, Hungary, and Italy (Bogaert et al., 2015; Bocquet et al., 2017; Bochenek et al., 2018; Videau et al., 2019).

Few countries have currently established a specific definition that includes the term “shortages” alone or in combination with the terms “medicines” or “drugs.” These include Belgium, Canada, Colombia, France, Hungary, Greece, Italy, Spain, and the US (**Table 4**).

Some countries have used logistics and market related terms, including Uruguay (declaration of interruption of sale or interruption of marketing), Argentina (medicines lacking), Croatia (disturbance on the medicines’ market), Norway (temporary disruption of a medicine’s marketing), Brazil, which uses the term “discontinuation” complemented with a term related to the temporality (definitive or unplanned or temporary), Italy (short supply), and Peru, which uses three terms “supply shortage,” “unavailable pharmaceutical products,” and “pharmaceutical product with limited supply” (**Table 4**).

The United States Food and Drug Administration (US FDA) has focused more on aspects related to scarcity and established a definition: “a period when the demand or projected demand for a medically necessary drug in the United States exceeds its supply” (CDER C for DE and research, 2018).

Bolivia, Chile, Croatia, Ecuador, Greece, Hungary, Norway, and Venezuela currently do not have an official definition of medicine shortage. These countries consider terms associated with medicine shortages but currently do not define the term medicine shortages (Bochenek et al., 2018). Finally, Venezuela differentiates scarcity as the situation in which there is insufficient quantity of a medicine to meet current demand and a supply shortage when the product is just not available (Ministerio de Salud Venezuela, 2017).

### Country Approaches

A new website at the European Medicines Agency (EMA) includes information about national legislation and local report mechanisms for 24 of the 30 European countries^1^. In South America, Brazil, Argentina, and Uruguay have regulations that make the reporting of situations that could potentially lead to shortages mandatory. EMA also have a regulation to report these cases.

Two qualitative studies have documented countries’ legislation and report mechanisms for three regions: Europe, Western Asia, and South America (ISAGS, 2017; Bochenek et al., 2018). As mentioned, this scoping review described one new region, North America (Center for Drug Evaluation and research C, 2018; Videau et al., 2019). In addition, it was possible to include information from 12 new countries: Australia, Bulgaria, Canada, Czech Republic, Denmark, Finland, Germany, Iceland, Romania, Sweden, United Kingdom (UK), and the US (All-party Pharmacy, 2015; Schwartzberg et al., 2017; The Society of Hospital Pharmacists of Australian, SHPA, 2017; CDER C for DE and research, 2018; Videau et al., 2019).

### Information Systems and Vigilance

Countries are taking actions regarding legislation and reporting systems in public websites (**Table 5**). Most of them are at the National Regulatory Agency level (28 countries), whereas others are at the Ministries of Health or National Health System level, including Colombia, Israel, Malta, Poland, Spain, Switzerland, UK, and Uruguay). Five countries were documented by Bochenek et al. in 2018 (Gulbis et al., 2013; Bochenek et al., 2018) where pharmacist professional organizations and other stakeholders are involved in medicines shortages reports. Finally, Canada has a website operated by a telecommunication company (**Table 5**).

**Table 5:** Frequency of update on the publicly available databases to report medicines shortages.

Frequency of updating	Country	Organization in charge of database	Access	Frequency of update	Mandatory Reporting
High	Australia	Therapeutics Goods Administration	https://apps.tga.gov.au/prod/MSI/search	Daily	Yes
	Belgium	Federal Agency of Medicines and Health Products	https://banquededonneesmedicaments.fagg-afmps.be/#/query/supply-problem-history/human	Daily (this is nominal frequency, which may be different)	Yes
	Canada	Bell Canada under contract with Health Canada	www.drugshortagescanada.ca/	Daily	Yes
	Czech Republic	State Institute for drug control	http://www.sukl.eu/dodavky-leciv-se-zamerenim-na-lecive-latky	Daily	Yes
	Latvia	State Agency of Medicines (SAMLV)	https://www.zva.gov.lv//?id=781&lang=&top=334&sa=673	Daily	Yes
	Portugal [1]	Portugal ANF - National Association of Pharmacies (ANF - Associação Nacional de Farmécias)	https://www.anfonline.pt/	Daily	Yes
	Sweden	Swedish medical products agency	https://lakemedelsverket.se/OVRIGA-SIDOR/Restnoteringar/	As soon as posible	Yes
	US	American Society of Health Systems Pharmacist - ASHP	https://www.ashp.org/drug-shortages/current-shortages	Daily	Not
Medium	Austria	Austrian Medicines and Medical Devices Agency (AGES MEA)	www.basg.gv.at/news-center/news/news-detail/article/uebersichtsliste-vertriebseinschraenkungen-986/	Weekly	
	Hungary	National Institute of Pharmacy and Nutrition	https://www.ogyei.gov.hu/temporary_discontinuation_of_sale_/	Weekly	Yes
	Italy	Italian Medicines Agency (AIFA—Agenzia Italiana del ármaco)	http://www.aifa.gov.it/content/carenze-e-indisponibilt%C3%A0	Weekly	Yes
	Malta [1]	Ministry for Health (CPSU—Central Procurement and Supplies Unit)	https://health.gov.mt/en/cpsu/Pages/POYC-OOS.aspx	Weekly	Yes
	Norway	Norwegian Medicines Agency	https://legemiddelverket.no/legemiddelmangel/legemiddelmangel-og-avregistreringer-2017-rad-til-apotek-og-helsepersonell	Weekly	Yes
	Slovakia	The State Institute for Drug Control (SUKL)	http://www.sukl.sk/en/inspection/post-authorization-quality-control/export-of-medicinal-products/list-of-medicinal-products-for-which-they-were-issued-decisions-not-to-allow-the-export-from-slovak-republic?page_id=4006	Weekly	Yes
	Spain [2]	Center for Information on Medicines Supply (in Spanish: Centro de Información sobre el Suministro de Medicamentos; CISMED)	http://www.portalfarma.com/Profesionales/consejoinforma/Paginas/Infarma-2016-CISMED.aspx	Weekly	Yes
	Switzerland [1]	Federal Office of Public Health	https://www.bag.admin.ch/bag/de/home/themen/mensch-gesundheit/biomedizin-forschung/heilmittel/sicherheit-in-der-medikamentenversorgung.html?_organization=317	Weekly	Yes
	Switzerland [2]	Federal Office for national economic supply (FONES)	https://www.bwl.admin.ch/bwl/de/home.html	Weekly	Yes
	Switzerland [3]	Swissmedic (Swiss Agency for Therapeutic Products)	https://www.swissmedic.ch/marktueberwachung/00135/00136/00140/00142/index.html?lang=de	Weekly	Yes
	Switzerland [4]	Martinelli Consulting Switzerland	www.drugshortage.ch	Weekly	Not applicable (private and voluntary but highly effective initiative)
	Turkey	Turkish Medicines and Medical Devices Agency: TMMDA (in Turkish: Türkiye Ilaç Ve Tibbi Cihaz Kurumu; TİTCK)	http://www.titck.gov.tr/	Weekly	Yes
Low	Argentina	National Administration of Medicines, Food and Medical Technology (ANMAT)	https://www.argentina.gob.ar/faltante-de-medicamentos	Twice a month	Yes
	Croatia	Croatian Health Insurance Fund	http://www.hzzo.hr/zdravstveni-sustav-rh/trazilica-za-lijekove-s-vazecih-lista/	Monthly	Yes
	France [1]	French Agency for Medicines Safety (ANSM – Agence Nationale pour la Sécurité du Medicament)	http://ansm.sante.fr/Mediatheque/Publications/Information-in-English	Yearly	Yes
	France [2]	National Council of the College of Pharmacists (Conseil national de l’ordre national des pharmaciens – CNOP)	http://www.ordre.pharmacien.fr/Le-Dossier-Pharmaceutique/Ruptures-d-approvisionnement-et-DP-Ruptures	Monthly	Yes
	Lithuania	State Medicines Control Agency (SMCA)	www.vvkt.lt	Biweekly	Unspecified
	Greece	National Organization for Medicines (EOF)	http://www.eof.gr/web/guest/eparkeia	Monthly (this is usual frequency)	Yes
	Ireland [1]	Irish Pharmaceutical Union (IPU)	https://ipu.ie/home/ipu-product-file/medicine-shortages/	Minimum monthly, but on demand based on completion of medicines shortages notification form	Yes
	Poland	Ministry of Health	http://www.bip.mz.gov.pl/legislacja/akty-prawne/obwieszczenie-ministra-zdrowia-z-dnia-10-stycznia-2017-r-w-sprawie-wykazu-produktow-leczniczych-srodkow-spozywczych-specjalnego-przeznaczenia-zywieniowego-oraz-wyrobow-medycznych-zagrozonych-brakiem/	At least bimonthly	Yes
Unspecified	Brazil	National Health Surveillance Agency (ANVISA)	http://portal.anvisa.gov.br/descontinuacao-de-medicamentos	As necessary (after every notification by MAH	Yes
	Bulgary	Bulgarian Drug Agency	http://www.bda.bg/bg/	As requiered	Not know
	Colombia [1] (may 2018 until now)	National Institute of Food and Medicines Surveillance (INVIMA)	https://www.invima.gov.co/desabastecimiento-de-medicamentos	As required	Yes
	Colombia [2] (2012 until april 2018)	Ministry of Health	https://www.minsalud.gov.co/salud/MT/Paginas/desabastecimiento.aspx	As required	Yes
	Denmark	Danish Medicines Agency	https://laegemiddelstyrelsen.dk/da/godkendelse/kontrol-og-inspektion/alvorlige-forsyningsvanskeligheder/#	As required	Yes
	Estonia	Estonian State Agency of Medicines	http://www.ravimiamet.ee/ulevaatlik-tabel-humaanravimite-tarneraskustest	As necessary (after every notification by MAH)	Yes
	Finland	The Finnish Medicines Agency - Fimea	https://www.fimea.fi/tietoa_fimeasta/ajankohtaista/saatavuushairiotiedotteet	As required	Yes
	Germany	Instituto Federal de Medicamentos y Dispositivos Médicos	https://www.bfarm.de/DE/BfArM/_node.html	As required	Yesit
	Israel	Ministry of Health	www.health.gov.il	According to the need	Yes
	Ireland [1]	UniPhar	www.uniphar.ie	As required	Yes
	Kosovo	Kosovo Medicines Agency	www.akppm.com	–	No
	Malta [1]	Ministry for Health (CPSU – Central Procurement and Supplies Unit)	https://health.gov.mt/en/cpsu/Pages/Items-Problematic-To-Source.aspx	As required	Yes
	Republic of Srpska, Bosnia and Herzegovina	The Agency for Medicinal Products and Medical Devices of Bosnia and Herzegovina (ALMBIH)	http://www.almbih.gov.ba/vijesti/	As soon as the ALMBIH is informed by the MAH about shortage of a given medicine	Yes
	Romania	National Agency for Medicines and Medical Devices	https://www.anm.ro/en/	As required	Yes
	Spain [1]	Spanish Agency of Medicines and Health Products (AEMPS), being part of the Spanish Ministry of Health	https://cima.aemps.es/cima/fichasTecnicas.do?metodo=buscarDesabastecidos	As required (whenever a shortage is detected)	Yes
	Slovenia	Agency for Medicinal Products and Medical Devices (JAZMP), and Health Insurance Institute of Slovenia	http://www.jazmp.si/fileadmin/datoteke/seznami/SFE/Prisotnost/Seznam_44_HUM_prenehanja_motnje.pdf www.cbz.si	Irregular	Yes
	United Kingdom	Specialist pharmacy service	https://www.sps.nhs.uk/	As needed	Yes
	Uruguay	Ministry of Public Health	https://tramites.gub.uy/ampliados?id=2659	As needed	Yes
	US [2]	Food and Drug Administration	https://www.accessdata.fda.gov/scripts/drugshortages/	Not specified	Not

Some of the main features of these systems include the frequency of updates, the obligation of pharmaceutical companies or marketing authorization holders to notify key stakeholder groups, and public institutions in charge of the database. To enhance our understanding of the characteristics of databases on medicine shortages in selected countries of Europe, Western Asia, North America, and Latin America, all databases have been divided into four groups, depending on frequency of their updating: high (daily), medium (weekly), low (less often than weekly), and unspecified (**Table 5**).

Most of the countries have only a national reporting system, where the Ministry of Health or the National Regulatory Agency manage the database and request mandatory reports. However, other countries have more than one system involved in the gathering of information, such as professional associations in France, Ireland, Malta, Portugal, Spain, and Switzerland (Bochenek et al., 2018). Finally, 12 of 40 countries have not reported databases on medicine shortage, six from Europe (Albania, Cyprus, Lichtenstein, Montenegro, Netherlands, Serbia), seven from South America (Guyana, Suriname, Ecuador, Bolivia, Paraguay, Peru, Venezuela), and Azerbaijan from Western Asia. None of the databases reported affected health conditions, or the possible impact caused by the reported episode.

Although all analyzed European countries have mandatory reporting systems, this scoping review revealed information systems with high frequency of shortages reports in countries, such as Australia, Canada, and the US. Countries of Latin America and Europe have low or unspecified update frequency of their medicines shortages websites on their (**Table 5**).

### Networks and Initiatives

In 2014, the South American Council for Health from UNASUR issued a declaration on “Access to medicines and problems of medicines shortage,” which stated that medicines shortage is a global and regional problem that manifests itself in diverse and changing ways, with various effects since there is insufficient information to determine the magnitude and features of the problem (ISAGS, 2017).

During 2016, several South American countries proposed to document the shortages situation of essential medicines in the region and to formulate strategies as part of the South American Institute of Government in Health (ISAGS UNASUR) actions for 2017. ISAGS UNASUR, together with the Andean Health Organization (Hipólito Unanue Agreement), undertook a study which described the situation in the member countries of the Andean and South American regions (ISAGS, 2017). An analysis of the medicines shortage situation of essential medicines was developed through the collection of information on decisions related to the problem, types of medicines identified by each country, identified causes, protocols of approach, solutions implemented, management and impact indicators, as well as limitations and experiences both at the country and regional levels that may be relevant to helping overcome the problem.

In 2016, the research collaboration initiative funded by the European Union was started. It is named COST CA15105—European Medicines Shortages Research Network—addressing supply problems to patients (Medicines Shortages) and it aims to stimulate and develop scientific research, as well as to propose solutions by end of 2020 (COST, 2015; COST, 2018). The COST CA15105 network encourages systematic sharing of information and research about shortages of medicines and nutritional products. It also aims to respond to the diverse interests of clinical and financial parties and patients’ quality of life, to achieve analytical clarity on the causes of shortages, to simulate appropriate decision making in manufacturing and the trade of medicines, to highlight legal and economic frameworks, to disclose disincentives in the supply chain, as well as to reflect on best coping practices to help ensure patients’ health is not compromised by ongoing shortages.

At the EU level, in 2016, the EMA and the Heads of Medicines Agencies (HMA) created an HMA/EMA Task Force on the Availability of Authorized Medicines for Human and Veterinary Use, aiming to provide strategic support and advice to tackle disruptions in the supply of human and veterinary medicines and to ensure their continued availability (EMA, 2019). The key priorities of the HMA/EMA Task Force included i) looking at ways to minimize supply disruptions and avoid shortages; ii) developing strategies to improve prevention and management of shortages caused by disruptions in the supply chain; iii) encouraging best practices within the pharmaceutical industry to prevent shortages; iv) improving sharing of information and best practices among EU regulatory authorities to better coordinate actions across the EU, and v) fostering collaboration with key stakeholders and enhancing communication of supply problems to EU citizens. A set of documents was published by EMA to support regulators involved in coordinating shortage situations due to good manufacturing practice (GMP) non-compliance. A public catalogue for shortages has been established by EMA^2^, which is designed to communicate clear information on shortages to patients, health care professionals, and other stakeholders.

## Discussion

### Studies Description

The studies included in this scoping review describe regional and countries contexts of medicine shortages and current perceptions of stakeholders, as well as their different perspectives. Indexed literature from the last 6 years presents important challenges, such as research on health and economic implications caused by supply disruptions and medicines shortages (Cousins, 2014; Steers, 2014; de Weerdt et al., 2017; Videau et al., 2019). More research efforts are needed to fully estimate the impact of medicine shortages on patients’ health especially in ambulatory care.

The descriptive cases reported from the US identified parenteral and hospital medicines with shortages episodes and their implications. Moreover, published studies for countries, including Brazil, help to estimate the health implications of shortages of essential antibiotics (Griffith et al., 2012; Galvao et al., 2013; McKeever et al., 2013; Goldsack et al., 2014; Taylor et al., 2016; Mazer-Amirshahi et al., 2017). Next, efforts are needed to compare the current situation between country and regional levels.

### Shortage Definitions and Global Context

Some ministries and health authorities have developed important initiatives to the timely identification of potential medicine shortages alongside initiatives to manage episodes of interruption in the supply chain (Bocquet et al., 2017; Schwartzberg et al., 2017; Administración Nacional de Medicamentos A y TMA, 2018; CDER C for DE and research, 2018; INVIMA, 2018).

De Weerdt et al. (2015a) identified some elements included in the definitions, i.e., “supply,” “delivery,” “availability,” “permanent discontinuation of drugs,” and “time frame,” to consider a uniform definition for drug shortages among European countries. Definitions from **Table 4** do not involve “delivery” and “availability” as these are not reflected in the current formal definitions. Instead, other common terms are included, such as “market” and “lack.”

Other countries, including France, Belgium, Italy, Spain, Brazil, and Colombia, have adopted definitions that include the perspective of shortage and some aspects related to supply chain management and market determinants. However, it is worth mentioning that the US FDA defines the problem of medicines shortage only from the perspective of scarcity and includes also a lead time. In addition, the US FDA, unlike other countries including Brazil and France, states that they will never ask a producer to make medicines or change the amount of medicines to be manufactured as solutions for dealing with drug shortages. They do not see this as their role (CDER C for DE and research, 2018).

A further finding is that a few countries have established a specific period into their definition to confirm the shortage situation: Australia, Belgium, France, Norway, and Uruguay (Bogaert et al., 2015; Bocquet et al., 2017; ISAGS, 2017; The Society of Hospital Pharmacists of Australian, SHPA, 2017; Bochenek et al., 2018). There are though some differences based on the perspective of the definition. Belgium, France, Norway, and Uruguay establish the shortage situation in a specific point of the supply chain, for instance, community pharmacies, or hospital pharmacies, or wholesalers. These countries have included periods for shortages, which varies from 72 h to 30 days (ISAGS, 2017; Bochenek et al., 2018). On the other hand, Australia defined a period of 6 months considering the lack of a medication in the whole country (The Society of Hospital Pharmacists of Australian, SHPA, 2017). Uruguay uses a period less than 30 days as an “interruption of marketing” and more than 30 days as a “declaration of interruption of sale” (ISAGS, 2017).

One main feature of a specific shortage situation is the identity of the involved medicine, e.g., simvastatin (as an individual medicine) or the class of medicines (statins, lipid modulators). Shortages can also refer to a certain pharmaceutical form (e.g., injectable, capsule, or lotion), administration route (e.g., parenteral or oral), or a certain concentration or package size. The way the product episode is defined—broadly or narrowly—depends on the purposes of the analysis and the availability of information.

It should be highlighted that in 2017, a set of two co-existing definitions had been proposed by the WHO. On the supply side: “A ‘shortage’ occurs when the supply of medicines, health products and vaccines identified as essential by the health system is insufficient to meet public health and patient needs. This definition refers only to products that have already been approved and marketed, to avoid conflicts with research and development agendas.” On the demand side: “A ‘shortage’ will occur when demand exceeds supply at any point in the supply chain and may ultimately create a ‘stockout’ at the point of appropriate service delivery to the patient if the cause of the shortage cannot be resolved in a timely manner relative to the clinical needs of the patient” (WHO, 2017). The needs of patients about essential medicines are an important link in these definitions.

The report of the Director General of the WHO presented at the last Assembly on global shortage of medicines and access to them in 2018 (A71/12) gives considerable relevance to situations that are recognized as a shortage of medicines, which coincide with those recently reported by South American countries (ISAGS, 2017; Organizaciòn Mundial de la Salud O, 2017). They show how different points of the medicines’ value chain are affected by concerns with demand, which are caused both by the lack of commercial attractiveness and by logistics, and supply factors that prevent having medicines in a timely manner.

In contrast, the title of the WHO’s report refers to the global scarcity of medicines, and throughout the document, the term “shortage” is being used (Organizaciòn Mundial de la Salud O, 2017). In general terms, the difference between the two terms (“scarcity of medicines” and “shortage of medicines”) can be minimal. However, it is worth specifying that “scarcity” refers only to those situations in which for a specific need the medication does not exist, whereas “shortage” covers those cases in which it is not possible to obtain the medicine in a timely manner. These latter problems are not solved by activities linked to research, development, and innovation.

Terms, such as “availability” and “affordability,” have also to be clearly distinguished to describe the shortage phenomena where these occur to avoid confusion and concentrate on appropriate activities to address the situation.

Regarding “availability,” at the jurisdiction/country level, this term usually implies that a medicine has marketing authorization and is marketed (can be bought or obtained from the health system). At the pharmacy level, this usually means that the medicine could be dispensed at demand or within a short period.

To contrast this, the term “affordability” refers to the extent the product is available at a reasonable price/cost for the patient or health care system considering the purchasing capacity of the individuals and/or the health system and does not endanger the financial sustainability of the purchasers. As mentioned earlier, this is particularly important for patients in LMICs where there are currently high co-payments. Affordability is also a growing issue in medium- to high-income countries especially with regard to new cancer medicines and those for orphan diseases with ever increasing prices (Howard et al., 2015; Godman et al., 2017; Godman et al., 2018; Kwon et al., 2018).

The shortage of medicines is related to two main activities planned by the WHO to be implemented between 2019 and 2023. These include i) research and development of medicines, and ii) supply chain management (States, 2019). Previous studies have reported essential medicines that need rapid solutions to address current concerns (ISAGS, 2017), i.e., the penicillins for syphilis and congenital syphilis.

The description of cases, the identified causes, and definitions of medicine shortages typically involve attributes that facilitate the identification of key solutions to address the main challenges of health conditions affected by the shortages. Currently, the specific actions described in the last version of WHO roadmap for 2019 to 2023 (States, 2019) do not discuss ways of approaching this and possible solutions to these cases, which is a concern.

We have highlighted the difficulty of building a simple typology of medicine shortages based on the multiple possible combinations of criteria. It may be more useful to define a checklist of relevant criteria that allows the characterization of the episode and to predict/estimate the potential impact/effects according to the likely magnitude and length of the shortage. This again will be researched further in the future.

### Causes and Characteristics of the Context That Determines the Identified Episode

The causes and context that define the shortage episodes or interruptions in the supply chain are different from other areas of policy that affect medicine availability, such as the non-availability of medicines without a marketing license, or those still in the research phase, or issues of affordability, leading to a lack of reimbursement or funding.

**Figure 5:**
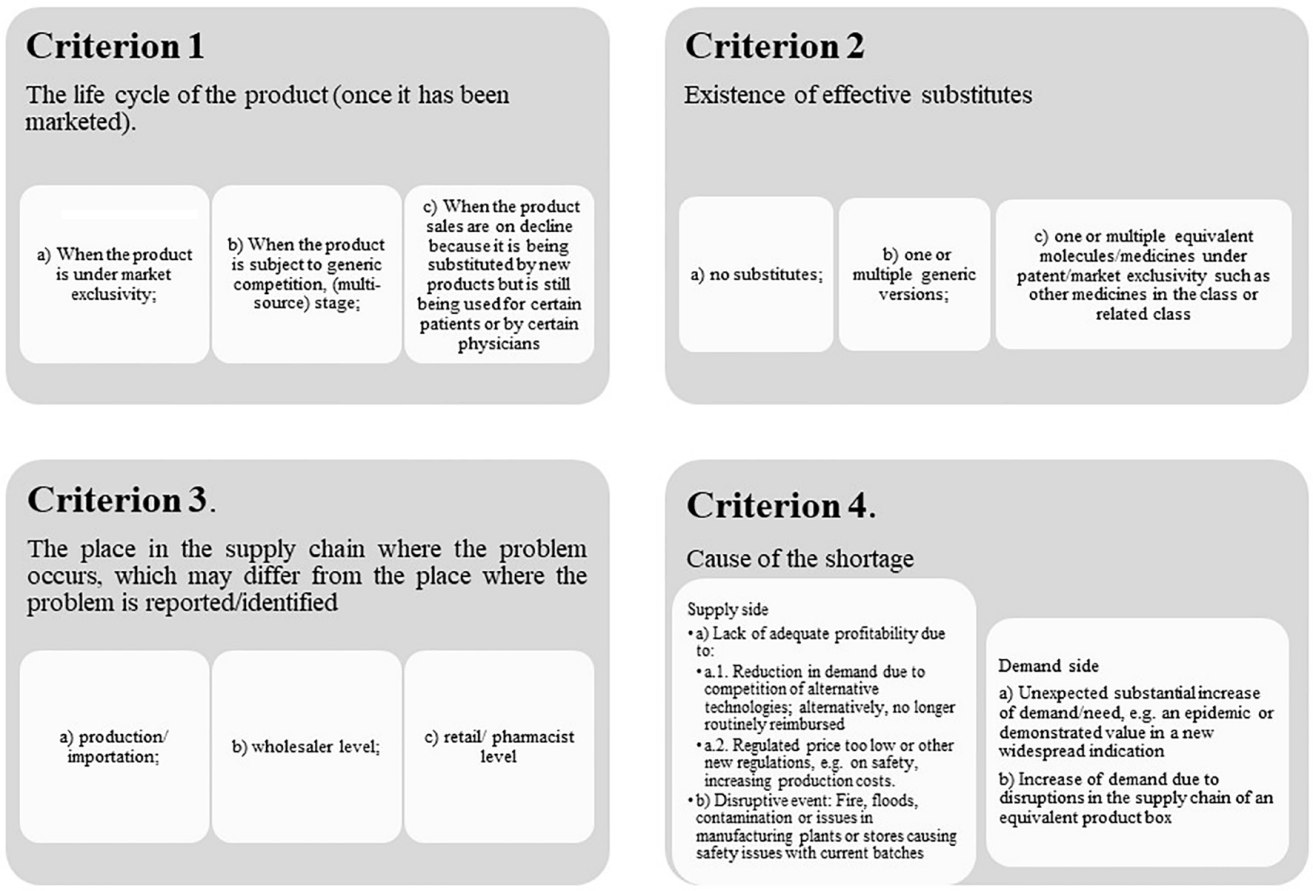
Proposed criteria for different approach on medicines shortage.

Overall, we believe medicine shortages can be defined according to several criteria (**Figure 5**).

In fact, medicine shortages might have multiple causes. Some of the causes might have a sequential cause-effect relationship, such as a reduction of future envisaged demand due to a reduction of the incidence of relevant diseases or reduced use due to the availability of new valued medicines, replacing existing ones, which will negatively impact on future profitability. This might cause the manufacturer to stop investing in the manufacturing process and disregard safety concerns, which may in turn lead to an increase in side effects and subsequent closure of the manufacturing plant.

Beyond an agreed single definition of medicine shortages, the concepts that could be developed must identify precisely the causes and points in the supply chain for which they apply. Topics, such as “shortage episode” could be more related to causes included in the market and policy dimensions (**Table 2**) or “interruption of supply chain episodes” for those situations caused or detected from the dimension of supply chain management.

### Global Framework

Once a shortage episode is identified and characterized, including the population and health condition affected, the appropriate pharmaceutical and health policy tools, as well as rational management of medicines and their stocks, could help identify the most effective and efficient available intervention to address the problem. This could include fast tracking authorization and importation of the medicine or an equivalent from another country or recommending physicians to prescribe a substitute medicine; alternatively, potentially increasing reimbursed prices where profitability is a concern. Coordination of national efforts and international cooperation is urgently needed on various levels to address this, including UNASUR, the Andean Health Organization-Hipólito Unanue Agreement, the European Union, and the WHO. We will be following this up in the future as well.

There have been issues in Europe with older cancer medicines when the originator company has sold their molecule to a generic manufacturer, and this was re-launched at a considerably higher price, prompting potential counter measures (Pagliarulo, 2017; Hawkes, 2017; Kahn, 2017; UK Department of Health and Social Care, 2016). Such activities will also be followed up, mindful of the need for manufacturers to make a profit for sustained supply.

Overall, we believe that specific strategies and solutions should be formulated at national and global levels. However, shortages must be clearly distinguished from scarcity. We believe this will lead to improved approaches to address episodes of medicine shortages and interruptions in the supply chain, as well as more pertinent solutions and their implementation to address current concerns.

There is a growing interest in international cooperation in the area of medicine shortages research, its prevention and ways to facilitate actions that provide a timely response. For South American countries, the development of a consensus to build or adapt a definition of medicines shortages demands the compilation of the countries’ definitions to generate a glossary related to logistics management and the availability of medicines. Some approaches, such as risk management and a panel of logistical indicators for medicines, can be adapted to each country according to its complexities, including the health system, epidemiological alerts, and geographical peculiarities. At the regional level, it has also been proposed to design a regional platform for horizon scanning and undertaking a pilot project to identify medicines that may be at risk of stock-outs, or possible withdrawals from the market, by reviewing the license status and reviewing databases related to imports and pharmaceutical market. No less important is to develop pilots for those priority medicines that are affecting the population health in the region.

### Approaches to Medicine Shortages

The proposed categorization of causes of medicines shortages can be used as a guidance to find potential solutions, as well as considering the supply stage of medicines, market competition, and possible therapeutic substitutes.

The Ministries and Health Authorities must consider which health conditions are affected by the shortage or supply chain interruption. This includes the proposed therapeutic use of the medicine and the availability of other therapeutic options, considering the optimal treatment for the disease or health condition involved within available resources.

Maybe the most critical causes of medicine shortages are those related to market aspects because these situations depend on the will of the producer, seller, or buyer, and the power relationship between each part. Some common situations are when a pharmaceutical company decides to suspend the production of a medicine, or the seller decides to modify the price of a specific pharmaceutical product, affecting its funding and supply. In view of this, possible solutions cannot just be focused on new possible innovations.

Likewise, countries initiatives should not just consist only of designing and implementing systems to compile information about cases of medicine shortages. The specific interventions should be moved forward to address potential shortages, as stated earlier.

Other approaches could include designing and implementing of risk management programs that allow the anticipation of shortage episodes as well as implementing activities, including horizon scanning to identify “programmed obsolescence” of a medicine based on market withdrawals or changes in the pricing of products. We have seen horizon scanning and budgeting successfully used in countries to better plan for the future, with Sweden and exemplar for other countries (Wettermark et al., 2010; Malmström et al., 2013; Eriksson et al., 2017; Godman et al., 2018), and these activities are likely to grow.

On the other hand, problems related to supply chain management are focused on logistics management and the level of the supply chain or network. These problems can be mitigated through the design of health care supply chains, with objectives that look to maximize the service level (availability and accessibility) and optimize the financial component, with recent changes in the public system in South Africa, a recent example among LMICs (Meyer et al., 2017). Ministries of Health should set key performance indicators for the country’s supply chain as well as implement improvements among the different agents in the supply chain over the different process including inbound and outbound transportation, fleet management, warehousing, materials handling, order fulfillment, logistics network design, inventory management, supply/demand planning, and the management of third-party logistics services providers (Council of Supply Chain Management Professionals (CSCMP), 2013). This will help reduce shortages in the future.

Some countries, such as France (Ordre National des Pharmaciens, 2015) have implemented actions focused on logistic aspects including defining a dead line to establish supply interruptions and length of shortage episodes. France also established a decree in 2012 to overcome the dysfunctions of the pharmaceutical distribution channel based on supply chain interventions, as well as introducing constraining and coercive measures for the various actors of the distribution channel. The decree defines shortages as the inability for a community or hospital pharmacy to deliver a medicine to a patient within 72 h if the medicine is currently not available, as well as possible therapeutic alternatives if needed, or which supply difficulties may lead to a risk of public health for patients, thereby creating an emergency. France established a procedure whereby all the actors of the medicine’s distribution channel should join forces to help address current concerns. The decree also established some regulatory obligations for pharmaceutical companies, including ensuring an appropriate and continuous supply of medicines to wholesalers to meet public and patients’ needs, implementing emergency call centers to inform agents about an anticipated or a current medicine shortage, informing the French authorities of emergency supplies, specifying the quantities and addresses, and informing of any risk concerns associated with any medicine shortage. The wholesaler must also declare its distribution territory, meet public obligations, have a selection of medicines at least nine tenths of commercialized pharmaceutical products in France, and ensure to deliver medicines within 8 h from any request (Ordre National des Pharmaciens, 2015; Bocquet et al., 2017). However, there are still challenges especially in the field of medicines with major therapeutic interest, and after 7 years of the introduction of the decree, there are still tensions between the different agents in the supply chain in France due to other causes not related to supply chain management (Biétry, 2018).

## Conclusions

Descriptions of medicines shortage extracted from the multiple studies included in this scoping review allowed the authors to extract main characteristics, as well as contexts and reasons of medicines shortage, which medicines are involved, definitions of shortages, countries information systems on shortage reports and alerts management, and finally any networks between countries and stakeholders’ initiatives.

We believe this is first time such a comprehensive study has been undertaken reporting the most representative description of the current medicine shortage situation across multiple countries and giving inputs regarding national and global health policies related to the most frequent reasons reported for medicine shortages. This includes the description of the most likely medicine classes with shortages, current national definitions, and related terms and available databases to report medicine shortages. The study also describes frameworks and approaches among countries to help address these issues in the future. Overall, we believe our findings can help with the development of definitions related to the shortage of medicines that can be universally applied to prevent future confusion.

Signals linked with unattended or affected health conditions because of shortages must also be documented to establish effective interventions from a health authority perspective to ensure patients’ health is not compromised. Indeed, policy makers require solutions that prevent those cases in which the population’s health is affected by episodes of medicine shortages and/or interruption in the supply chain.

As part of this, we believe it is necessary to develop a consensus to build or adapt definitions of shortage and scarcity, as well as identifying all local definitions, to generate a glossary related to logistics management and the availability of medicines to facilitate aggregation of data from different sources and to better manage future situations. We have started this process with this article.

We also believe it is important to emphasize that for those cases where shortages are due to logistical and supply factors, and it is not possible to have the medicines in a timely manner, that potential ways forward are not related to improving research, development and innovation as some bodies are proposing. This is a very different situation, especially if alternative solutions are available including importation, as well as permitting increased prices if pertinent along with advocating and documenting possible alternatives, and these must be explored first.

Overall, we believe it would also be useful to identify options and best practices on how to address the most common causes of shortages and share these among all countries to provide future direction. These are research topics for the future building on this review across countries.

Our findings show that part of the problem of shortages is focused on provision, especially those cases related to the permanent suspension of production or voluntary suspension, due to, for instance, to the lack of commercial interest, either by low demand, end of the patent, price regulations, use of compulsory licenses and/or the introduction of new medicines. Regarding suspending commercialization, it is important to review specific cases that raise considerable public health concerns, such as antineoplastic and antibiotics, to suggest potential ways to overcome the situation. We have started this process and will continue.

## Limitations

Although the findings presented in this study allows us to make comparisons between countries related with, for instance, national definitions and involved medicines, there are other attributes that may well broaden the situation which we have not discussed. These can be addressed going forward.

## Recommendations

Further research is needed to fully assess the clinical and economic impact of medicine shortages at different jurisdictional levels, including both hospital and ambulatory care levels. Additionally, cross-country and regional studies will help to identify current essential medicines supply disruptions and shortages episodes, which need prompt solutions.

## Author Contributions

AA and EV conducted the study and prepared the first draft of the article and the literature review. JR brought methodologic inputs and assessed preliminary results. JR, BG, and TB commented on and contributed to subsequent iterations on the manuscript.

## Conflict of Interest Statement

TB is an active member (Action Chair) of COST CA15105—European Medicines Shortages Research Network—addressing supply problems to patients (Medicines Shortages) funded by the European Union.

The remaining authors declare that the research was conducted in the absence of any commercial or financial relationships that could be construed as a potential conflict of interest.

## Footnotes

1 European Medicines Agency. Medicine shortages. https://www.ema.europa.eu/en/human-regulatory/post-authorisation/medicine-shortages

2 European Medicines Agency—https://www.ema.europa.eu/en/human-regulatory/post-authorisation/medicine-shortages/shortages-catalogue
