# Supplementary material for: Medicine Shortages: Gaps Between Countries and Global Perspectives
Source: Front Pharmacol. 2019 Jul 19;10:763. doi: 10.3389/fphar.2019.00763 (PMC6658884; doi:10.3389/fphar.2019.00763)
Supplement: Supplementary file 1 [file DataSheet_1.pdf]

Supplementary Material

Southamerican countries` 2017 Survey form

ISAGS is giving advance to actions foreseen in the Annual Operational Plan 2017, as you know we begun the study of essential medicines with risk of shortages in South America.

We kindly request the focal points of UNASUR's GAUMU Group, to fill out the enclosed survey instrument and its annex.

The contact details of the medicines focal points could be forwarded to us in order to contact them and establish direct communication.

We reiterate the importance of having the contributions of focal points and members of the GAUMU Group to advance with the development of the study.

We appreciate your management and we will be attentive to your responses.

1) 1.If the Ministry of Health or the National Regulatory Agency of medicines recognizes a definition for shortages and / or shortages of medicines please register it below and indicate the source normative.

2) Groups of medicines that have presented shortages and / or shortages. A review of previous literature in databases of biomedical publications in recent years as well as in other media (advanced search - Google search engine) allowed to identify the most frequent pharmacotherapeutic groups and drugs, indicated in the Annex. Please enter the requested information.

3) Are there any other medicines for which a shortage or shortage situation has been identified? Specify them below.

4) List the possible causes that have generated situations of shortages or shortages of the medicines listed in numerals 2 and 3.

5) Does the Ministry or those involved have a process / protocol to address situations of shortages or shortages of medicines? State response with (X) in front of one of the two response categories.
Yes ___ No ___

6) If the answer to the previous number is "Yes", describe the process / protocol, if applicable, indicate the source and the sources of web consultation about it and its results

7) Describe the type of solutions implemented so far from the Ministry or related instances to overcome the situations of shortages or shortages presented.

8) Does the Ministry or related entities have indicators about processes / protocols implemented and situations of shortages or shortages overcome? State response with (X) in front of one of the two response categories.

Yes ___

No ___

9) If the answer to the previous paragraph is "Yes", describe indicators about processes / protocols implemented in situations of shortages or shortages overcome.

Ex. - Belgium: *Source: De Weerdt E, De Rijdt T, Simoens S, Casteels M, Huys I. Time spent by Belgian hospital pharmacists on supply disruptions and drug shortages: An exploratorystudy. PLoSOne. 2017 Mar 28;12(3):e0174556. doi: 10.1371/journal.pone.0174556.eCollection 2017. PubMed PMID: 28350827; PubMed Central PMCID: PMC5370124.*

10) Does the Ministry or related bodies have impact indicators on situations of shortages or shortages? State response with (X) in front of one of the two response categories.

Yes ___ No ___

11) If the answer to the previous paragraph is "Yes", describe indicators of impact on situations of shortages or shortages overcome.

12) Describe what type of limitations you have faced during the reporting of reported cases.

13) What medicines have been affected in your supply because of the limitations described above?

14) Describe successful experiences in overcoming drug shortages or shortages at the country level.

15) Describe successful experiences in overcoming drug shortages or shortages at the regional level.

16) on the experience of your country, list possible useful ways at the regional level to solve situations of shortages and / or shortages of medicines.

Ex: *Source: Schwartzberg et al. Drug shortages in Israel: regulatory perspectives, challenges and solutions. Israel Journal of Health Policy Research (2017) 6:17 DOI 10.1186/s13584-017-0140-9*
